# High-angular-sensitivity X-ray phase-contrast microtomography of soft tissue through a two-directional beam-tracking synchrotron set-up

**DOI:** 10.1107/S1600577524005034

**Published:** 2024-07-15

**Authors:** Carlos Navarrete-León, P. Stephen Patrick, Adam Doherty, Harry Allan, Silvia Cipiccia, Shashidhara Marathe, Kaz Wanelik, Michela Esposito, Charlotte K. Hagen, Alessandro Olivo, Marco Endrizzi

**Affiliations:** a Department of Medical Physics and Biomedical Engineering, University College London, Malet Place, Gower Street, LondonWC1E 6BT, United Kingdom; b Centre for Advanced Biomedical Imaging, Division of Medicine, University College London, Paul O’Gorman Building, 72 Huntley Street, LondonWC1E 6DD, United Kingdom; c Diamond Light Source Harwell Science and Innovation Campus, Fermi Avenue DidcotOX11 0DE United Kingdom; Tohoku University, Japan

**Keywords:** phase-contrast imaging, tomography, bioimaging

## Abstract

Two-directional beam-tracking (2DBT) is a method for phase-contrast imaging and tomography that uses an intensity modulator to structure the X-ray beam into an array of independent circular beamlets that are resolved by a high-resolution detector. It features isotropic spatial resolution, provides two-dimensional phase sensitivity, and enables the three-dimensional reconstructions of the refractive index decrement, δ, and the attenuation coefficient, μ. In this work, the angular sensitivity and the spatial resolution of 2DBT images in a synchrotron-based implementation is reported. In its best configuration, angular sensitivities of ∼20 nrad and spatial resolution of at least 6.25 µm in phase-contrast images were obtained. Exemplar application to the three-dimensional imaging of soft tissue samples, including a mouse liver and a decellularized porcine dermis, is also demonstrated.

## Abstract 2

X-ray phase-contrast microtomography is demonstrated using two-dimensional amplitude modulation of the beam. The implementation is characterised in terms of angular sensitivity and spatial resolution, and its application to the three-dimensional imaging of biological soft tissues is shown.

## 1. Introduction

X-ray phase-contrast tomography (XPCT) is a non-destructive imaging technique that enables the 3D visualization of materials and tissues composed of low-*Z* elements. Through generating contrast also from the phase shift induced in the X-ray wavefront, this technique allows for the visualization of details otherwise undetectable by using a conventional, attenuation-based approach to generating image contrast (Paganin & Pelliccia, 2021 ▸). Notable examples have been demonstrated in the biomedical field using synchrotron radiation, including brain (Pinzer *et al.*, 2012 ▸; Töpperwien *et al.*, 2020 ▸), lung (Xian *et al.*, 2022 ▸; Reichmann *et al.*, 2023 ▸), kidney (Zdora *et al.*, 2020 ▸; Walsh *et al.*, 2021 ▸), breast (Zhao *et al.*, 2012 ▸; Baran *et al.*, 2018 ▸; Peña *et al.*, 2023 ▸), lymph node (Schwarzenberg *et al.*, 2022 ▸) and oesophagus (Hagen *et al.*, 2015 ▸) imaging, amongst others.

Several methods have been developed at synchrotron radiation facilities for XPCT including crystal-based interferometric methods, propagation-based imaging methods, analyser-based imaging methods, grating-based interferometric methods, speckle-based imaging methods and non-interferometric mask-based methods (Bonse & Hart, 1965 ▸; Momose *et al.*, 1996 ▸; Pfeiffer *et al.*, 2006 ▸, 2008 ▸; Snigirev *et al.*, 1995 ▸; Nugent *et al.*, 1996 ▸; Chapman *et al.*, 1997 ▸; Momose, 2003 ▸; Morgan *et al.*, 2012 ▸; Berujon *et al.*, 2012 ▸; Olivo *et al.*, 2001 ▸; Morgan *et al.*, 2011 ▸; Vittoria *et al.*, 2015 ▸). We refer to some recent reviews for a comprehensive list of the different milestones and their relative advantages (Momose, 2020 ▸; Quenot *et al.*, 2022 ▸).

Here we focus on two-directional beam-tracking (2DBT), which belongs to the category of non-interferometric mask-based methods. The method was first proposed by a patent in the mid-1990s (Wilkins, 1995 ▸) and it shares similarities with the Shack–Hartmann wavefront sensor. The earliest demonstrations of this approach include the use of an array of refractive micro lenses (Mayo & Sexton, 2004 ▸), a 1D structure as phase grating (Takeda *et al.*, 2007 ▸), and a 2D transmission grating with Fourier analysis (Wen *et al.*, 2010 ▸). In our work, we use a high-*Z* amplitude modulator to structure the beam into an array of independent beamlets that are then resolved by a high-resolution detector. The modulator is placed before the sample, contrary to other implementations (Provinciali *et al.*, 2021*a* ▸,*b* ▸), meaning that all photons reaching the sample contribute to image formation, limiting the absorbed dose.

In this approach, the radiation field intensity modulation, coupled with a dedicated data analysis methodology, provides a way to measure how the sample affects the intensity and position of each X-ray beamlet. A shift in their position is interpreted as a refraction effect, which is related to the first derivative of the phase shift imposed by the sample. A change in intensity is interpreted as attenuation of the X-ray beam. A broadening of the beamlets is linked to the ultra-small-angle scattering signal (Vittoria *et al.*, 2015 ▸; Dreier *et al.*, 2020 ▸), also known as X-ray dark-field imaging.

This approach provides aperture-driven (Diemoz *et al.*, 2014 ▸; Hagen *et al.*, 2018 ▸; Esposito *et al.*, 2022 ▸) and isotropic (Navarrete-León *et al.*, 2023*a* ▸) spatial resolution; and, with dedicated mask designs alongside efficient acquisition schemes, scanning time can be improved for optimal acquisition (Lioliou *et al.*, 2022 ▸, 2023 ▸), and fly scan data acquisition schemes. The method was recently demonstrated in a compact laboratory set-up (Navarrete-León *et al.*, 2023*b* ▸), within a small (<1 m) footprint and by using a low power (10 W) source.

Here we report on an implementation that made use of synchrotron radiation, and that we found suitable for high-sensitivity measurement of phase gradients, providing excellent contrast for the visualization of the morphology in soft tissue samples. We focus on imaging through refraction and attenuation contrast, as we found these channels were sufficient for obtaining high-quality three-dimensional images of the soft tissues investigated and for characterizing the sensitivity of our setup under a wide range of configurations. We report on the angular sensitivity, characterized as a function of exposure time and system geometry, as well as the spatial resolution, estimated with Fourier ring correlation on tomographic reconstructions. We demonstrate exemplary application to the three-dimensional imaging of soft tissue samples, including both a formalin-fixed sample of mouse liver, as well as a decellularized, iodine-stained porcine dermis. For both biological samples, phase-contrast imaging enhanced the visibility of key physiological features above attenuation-based images acquired with equivalent X-ray exposure.

## 2. Materials and methods

### 2.1. 2DBT X-ray set-up

**Figure 1:**
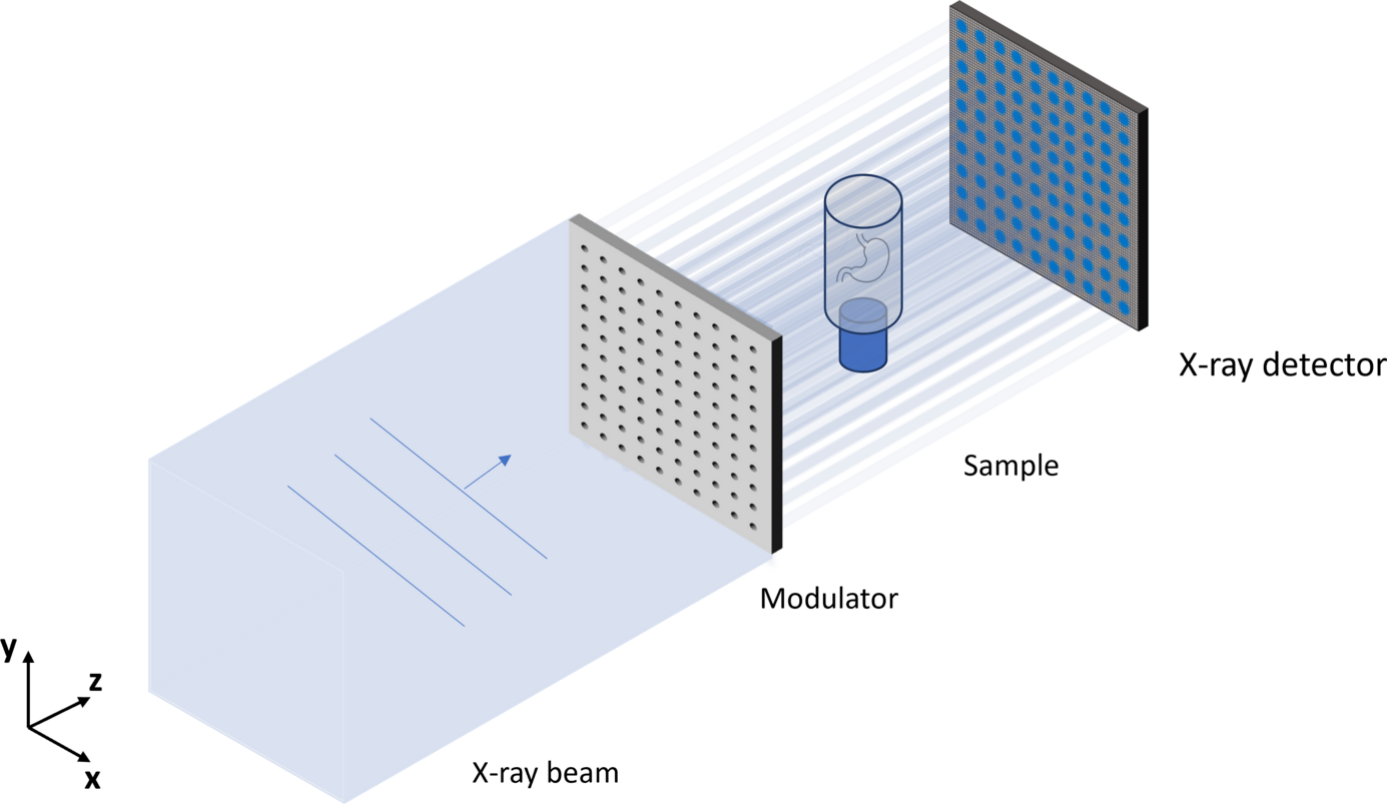
Schematic diagram of the two-directional beam-tracking experimental set-up.

The XPCT set-up is presented in Fig. 1 ▸. The experiments were carried out at Diamond Light Source beamline I13-2. The angular sensitivity measurements were made with a mean energy of 16 keV from a filtered pink beam with a silicon mirror and filters of 1.34 mm pyrolytic graphite, 1.4 mm aluminium and 0.042 mm niobium. For the biological specimens, the mean energy was increased to 27 keV to reduce sample damage by changing to a platinum mirror and filters of 1.34 mm pyrolytic graphite and 3.2 mm aluminium.

The sample was placed roughly 221 m from the source, and the modulator was placed 15 cm upstream of the sample. The modulator is fabricated with laser-ablation from a 100 µm-thick tungsten foil (Goodfellow), and has a period of 50 µm. The apertures have a conical shape with diameters of 15 µm in the front and 30 µm in the back. The detector is a pco.edge 5.5 camera coupled to a scintillator-objective combination with an effective pixel size of 2.6 µm × 2.6 µm and a field of view of 6.6 cm × 5.6 cm.

### 2.2. Planar imaging and angular sensitivity measurements

The angular sensitivity was assessed with a custom-built phantom composed of soda-lime glass microspheres of 50 µm diameter (Fischer Scientific, monodisperse) embedded in wax and polyethylene foam. To study the sensitivity as a function of the object-to-detector distance (*z*_od_), the sample was imaged at the following distances, *z*_od_ = {2.5, 7.5, 17.5, 37.5, 77.5} cm, by moving the detector. The sample was moved in a 10 × 10 grid with steps of 5 µm in the *xy* plane. At each sample position, 10 × 0.1 s frames were acquired, and flat and dark images were taken before and after each scan. The frames were used to assess sensitivity as a function of the exposure time. The assessment of the angular sensitivity during imaging was carried out by calculating the mean and standard error of the standard deviation of the measured refraction angles in an area without the sample, for which eight different windows of 5 × 40 pixels were used.

### 2.3. Tomography of unstained and stained ex-vivo tissues

Two biological samples were imaged: a mouse liver and a hernia mesh, consisting of decellularized porcine dermis. The liver was fixed in 4% paraformaldehyde for 24 h upon dissection from a two-month old C57BL/6 mouse (Charles River Laboratories), then stored in 0.9% saline. The decellularized porcine dermis (Xenmatrix^TM^, Bard) was stained in 3% Lugol’s iodine solution (Scientific Laboratory Supplies) in phosphate buffered saline (Gibco) for 24 h before storage in 0.9% saline (Peña *et al.*, 2023 ▸). Both liver and decellularized dermis were prepared for imaging by embedding in 1% agar (Thermo Scientific Chemicals). The samples were between 3.1 and 3.8 mm wide and they were scanned by acquiring 1200 projections while rotating over 180° in a fly scan fashion with an exposure time of 0.15 s per projection. This was repeated at different modulator sub-pitch displacements to increase sampling. The modulator was raster-scanned in 8 × 8 positions, by using 6.25 µm displacements both in *x* and *y*. This led to a total exposure time of 1200 × 8 × 8 × 0.15 s = 3.2 h for each sample. Flat and dark images were acquired at each modulator position, before and after rotating the sample. Note that this is different from planar imaging, in which it was the sample that was moved. For tomography acquisitions we move the modulator to allow the sample to be rotated in a fly scan mode. This reduces the total number of movements and allows for a substantial reduction of overhead scanning time. The detector was placed 128 cm away from the sample to further increase the angular sensitivity, which was also assessed for this configuration with eight different windows of 8 × 8 pixels.

### 2.4. Data analysis

The transmission, refraction in *x* and refraction in *y* images were obtained by selecting a window of 20 × 20 pixels around each beamlet and comparing the intensities with [*I*_s_(*x*, *y*)] and without [*I*_0_(*x*, *y*)] the sample in the beamlet. The transmission was calculated by dividing the sum of the intensities in the windows: *t* = 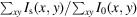, and the two refraction images by measuring the displacements (Δ*x*, Δ*y*) between the beamlets with a subpixel cross-correlation algorithm (Guizar-Sicairos *et al.*, 2008 ▸). This was performed for all images acquired at each sample or modulator position, which were then stitched to obtain an image with higher sampling (Navarrete-León *et al.*, 2023*b* ▸).

Assuming small refraction angles and under a geometrical optics approximation, the refraction angle, α_*xy*_, is related to the displacements Δ*x* and Δ*y* and the orthogonal gradients of the phase shift, 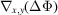, by
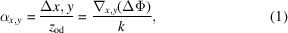
where *z*_od_ is the object-to-detector distance and *k* is the wavenumber. This allows us to obtain the phase shift ΔΦ by integrating the two gradients through a Fourier space method (Kottler *et al.*, 2007 ▸).

The retrieved quantities *t* and ΔΦ are linked to integrals along the photon path of the linear attenuation coefficient (μ) and the real part of the refractive index (δ) in the following way,
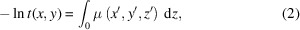

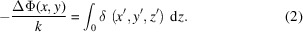
Therefore, for the biological specimens, volumes of μ and δ were obtained from the projections taken at different viewing angles using the filtered back projection (FBP) implementation of the *Astra Toolbox* (van Aarle *et al.*, 2015 ▸).

For both the μ and δ volumes, the spatial resolution of slices in the three orthogonal planes was estimated using an implementation of Fourier ring correlation (FRC) (Nieuwenhuizen *et al.*, 2013 ▸) provided as part of the BIOP *ImageJ* plugin (Herbert & Burri, 2016 ▸). Independent inputs were provided to the algorithm by reconstruction of two volumes, each using half of the available projections. To reduce the noise of the FRC estimate, the resultant curves of five adjacent representative slices from the middle of the volume were averaged. Resolutions are stated using the 3σ criterion, expressing the spatial frequency at which the FRC curve exceeds by three standard deviations the expected correlations within the random background noise (Van Heel & Schatz, 2005 ▸).

## 3. Results and discussion

### 3.1. Planar imaging and angular sensitivity

**Figure 2:**
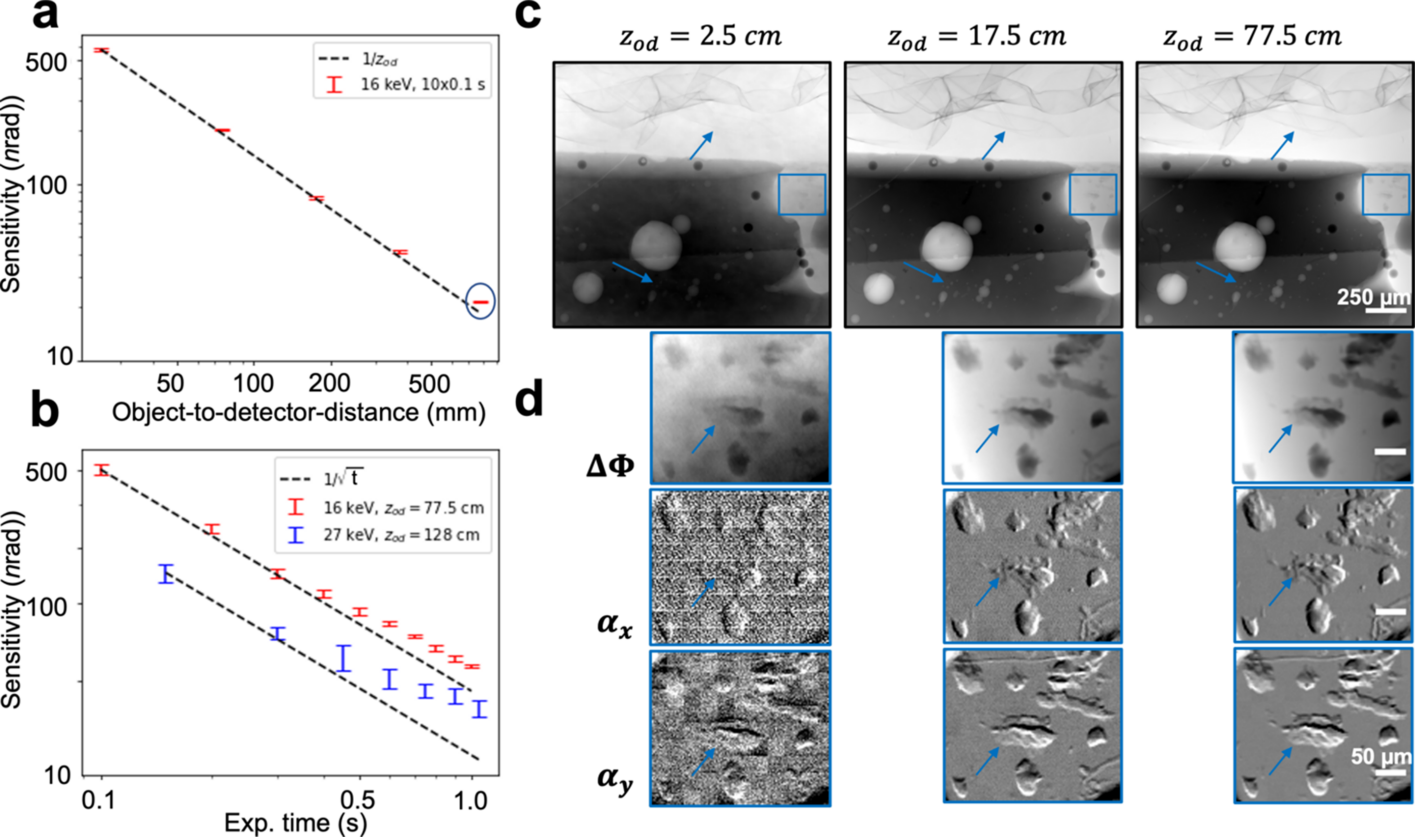
Angular sensitivity of the method as a function of (*a*) object-to-detector distance and (*b*) exposure time for different system configurations. (*c*) Phase images of the phantom (polyethylene foam, and microspheres and air bubbles embedded in wax) are shown for increasing object-to-detector distances. The improvement in angular sensitivity reveals interfaces in the foam and small bubbles in the wax substrate, as pointed out by the arrows. (*d*) An inset in the phase image is shown, along with the two refraction images, for further demonstration of thin wax deposits on the substrate being unveiled with increasing sensitivity.

The results from the angular sensitivity measurements are reported in Fig. 2 ▸. The sensitivity is shown for different object-to-detector distances [Fig. 2 ▸(*a*)] and for an increasing number of integrated frames for both energy configurations [Fig 2 ▸(*b*)]. We observe that the smallest resolvable angle decreases proportionally with increasing propagation distance (∝ 1/*z*_od_) within the range of propagation distances explored, as expected from the geometrical optics approximation used in equation (1). We note a small deviation from this trend at 77.5 cm of propagation distance [circled data point in the bottom right corner of Fig. 2 ▸(*a*)] and we observe that it is associated with a small decrease in visibility, from 85% to 82%. Longer propagation distances offer a further increase in the angular sensitivity; however, the angular sensitivity is expected to increase at a relatively slower rate beyond this point. In addition, we also observed that, when integrating a few frames, the sensitivity goes inversely with the square root of the exposure time (*t*^1/2^). This is the expected trend from Poisson statistics, indicating that this is the dominant noise source up to accumulations of 400 ms; beyond this point, additional noise sources become significant in limiting the smallest measurable refraction angle. The smallest angle with a mean energy of 16 keV was measured with a combination of *z*_od_ = 77.5 cm and 10 × 0.1 s frames, for which we report an angular sensitivity of 21.6 ± 0.2 nrad. In the conditions for tomographic imaging at 27 keV, the angular sensitivity benefits from the increased propagation distance of *z*_od_ = 128 cm, and we measured 35 ± 2 nrad with only 1 × 0.15 s frame.

The effect of increasing angular sensitivity on image quality can be observed in Fig. 2 ▸(*c*) and 2(*d*), where the integrated phase images are presented along with both refraction images of the smaller, highlighted region of interest. The increasing angular sensitivity unveils various interfaces in the foam and small bubbles in the wax substrate, as pointed out by the arrows in Fig. 2 ▸(*c*). This is further demonstrated with the insets in Fig. 2 ▸(*d*), where the two refraction images show increasingly lower noise levels as propagation distance is increased, which in this case reveals thinner deposits of wax on the substrate as angular sensitivity increases.

### 3.2. Tomography of biological soft tissues

**Figure 3:**
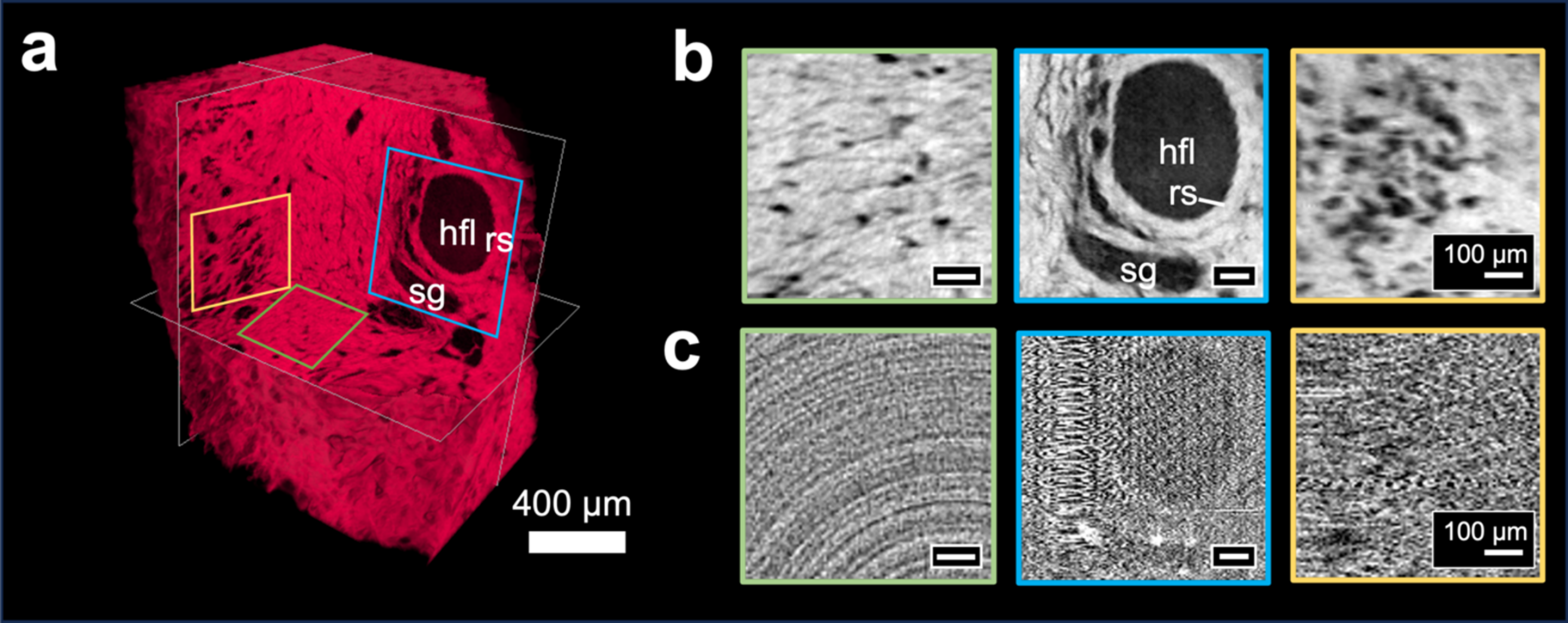
X-ray phase-contrast and attenuation tomography of decellularized dermis. (*a*) 3D render of phase-contrast tomography showing perpendicular cross-sections of (*b*) phase contrast and (*c*) attenuation. The following anatomical features were identified: hair follicles lumen (hfl), root sheath (rs) and sebaceous gland (sc).

**Figure 4:**
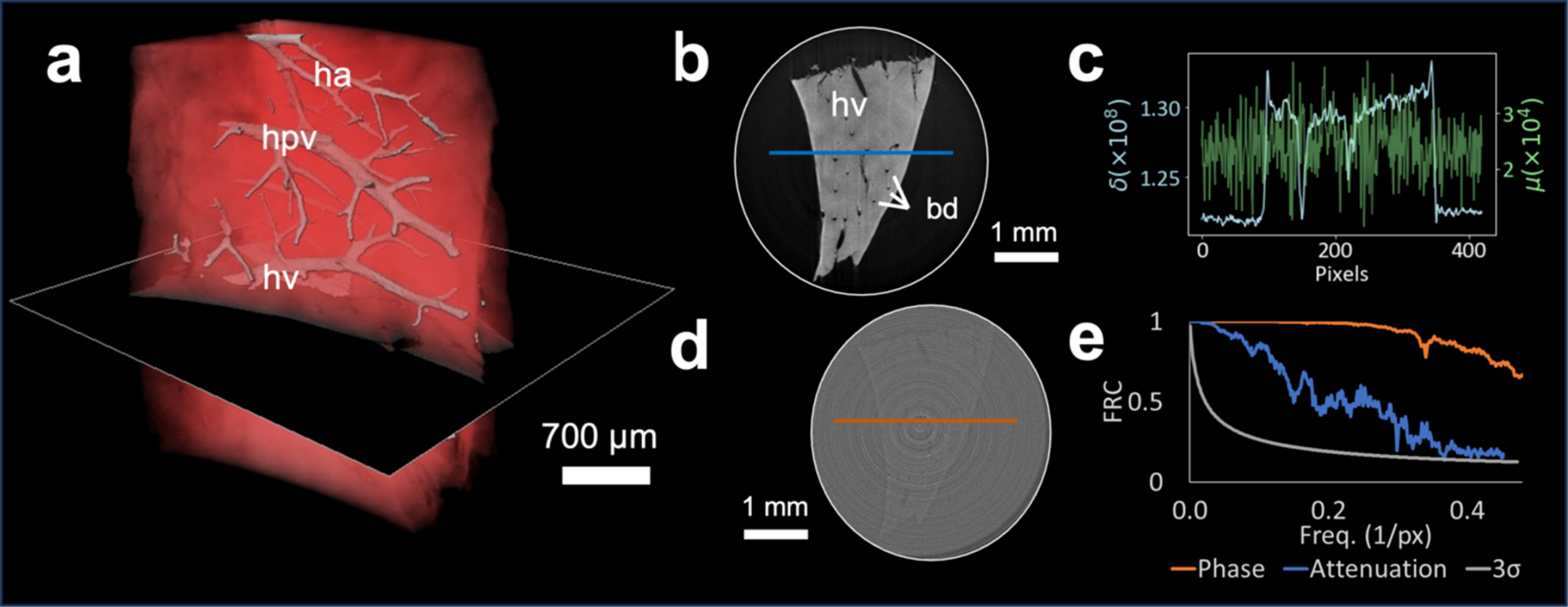
X-ray phase-contrast and attenuation tomography of mouse liver. (*a*) 3D rendering with phase-contrast tomography with an axial cut shown for phase (*b*) and attenuation (*d*) channels. (*c*) Line profile of the signal through indicated liver cross sections of (*b*) and (*d*) showing improved contrast to noise ratio in the phase contrast. (*e*) Fourier ring correlation curve calculated from the two independent reconstructions of (*b*) and (*d*) showing an increased resolution for phase contrast. The following anatomical features were identified with phase-contrast tomography: hepatic portal vein (hpv), hepatic vein (hv) and hepatic arteries (ha).

The attenuation and phase contrast tomographic reconstructions of the decellularized dermis and liver tissue samples are presented in Figs. 3 ▸ and 4 ▸, respectively. The significant increase in contrast-to-noise ratio achieved by means of phase contrast is evident across all slices and for both stained (Fig. 3 ▸) and unstained (Fig. 4 ▸) samples. While the image quality enhancement from phase contrast was particularly evident in the unstained (*i.e.* poorly absorbing) liver tissue, even tissue optimized for attenuation-based imaging using an iodine stain still showed notable improvement with phase contrast. For the stained sample of the decellularized dermis, while the lumen of the hair follicles (hfl) was discernible in both phase- [Figs. 3 ▸(*a*) and 3(*b*)] and attenuation-based images [Fig. 3 ▸(*c*)], phase contrast improved the clarity of the structure of the hair follicles including the layers of the root sheath (rs), sebaceous gland (sc) and fibre alignment around the dermic sheath [Figs. 3 ▸(*a*) and 3(*b*)]. In terms of physiological features identifiable in the liver sample, phase contrast gave clear visualization of the hepatic portal vein (hpv), the hepatic veins (hv), the hepatic arteries (ha) and the bile ducts (bd) [Figs. 4 ▸(*a*) and (*b*)], while these were not discernible on the attenuation-based images [Fig. 4 ▸(*d*)].

Fig. 4 ▸(*e*) displays the FRC curves obtained from the unstained liver tissue axial slices, as illustrated in Figs. 4 ▸(*b*) and 4 ▸(*d*). The attenuation curve intersects the threshold at 0.4 px^−1^, indicating a spatial resolution of 16 µm. Meanwhile, the phase-contrast curve fails to intersect with the threshold, suggesting that in this case the spatial resolution is sampling limited and is at least equal to the sampling pixel size of 6.25 µm. We note that the FRC curves in the orthogonal cross-sections showed comparable trends. We interpret the disparity between the FRC curves obtained through the attenuation- and phase-contrast tomography as a consequence of the noise and contrast dependence inherent in the FRC resolution metric. High spatial frequency image features are unable to surpass the noise threshold in the noisier, low-contrast attenuation volume, whereas the much higher signal-to-noise ratio achieved with phase contrast allows for separating even the smallest features from the background. We also note that by splitting the projection dataset to obtain independent volumes, the angular tomographic sampling has also been halved to 600 projections. Although the full dataset was largely oversampled in terms of viewing angles, the halved dataset is undersampled with respect to the Nyquist sampling theorem for samples between 500 and 600 pixels of width. As such, the FRC result should still be considered a conservative estimate of the achievable resolution.

## 4. Conclusion

We have here studied the angular sensitivity and spatial resolution in images obtained through a 2DBT synchrotron set-up and shown the potential of the method for volumetric imaging of soft tissues with poor attenuation contrast. We report angular sensitivities of ∼20 nrad at 1 s exposure time, 77.5 cm of propagation distance and 16 keV mean energy; and ∼35 nrad at 150 ms, 128 cm and 27 keV mean energy. Our results indicated that the geometrical-optics approximation used by the phase-retrieval algorithms is well satisfied within 1 m of propagation distance. We have also shown sub-aperture spatial resolution in phase-contrast tomography, which was observed to be limited by sampling to a factor 2.4× better than the apertures in the modulator. These results provide a basis for future experimental designs with the 2DBT method, especially for identifying optimal trade-offs between angular sensitivity, spatial sampling and acquisition time. They also provide a basis for comparison with similar imaging methods.
